# A Qualitative Exploration of Community Ownership of a Maternity Waiting Home Model in Rural Zambia

**DOI:** 10.9745/GHSP-D-20-00136

**Published:** 2020-09-30

**Authors:** Constance P. Fontanet, Rachel M. Fong, Jeanette L. Kaiser, Misheck Bwalya, Thandiwe Ngoma, Taryn Vian, Godfrey Biemba, Nancy A. Scott

**Affiliations:** a Department of Global Health, Boston University School of Public Health, Boston, MA, USA.; b Department of Research, Right to Care Zambia, Lusaka, Zambia.; c School of Nursing and Health Professions, University of San Francisco, San Francisco, CA, USA.; d National Health Research Authority, Pediatric Centre of Excellence, Lusaka, Zambia.

## Abstract

**Context:**

Ownership is an important construct of sustainability for community-based health programming, though it is often not clearly defined or measured. We implemented and evaluated a community-driven maternity waiting home (MWH) model in rural Zambia. We engaged stakeholders at all levels and provided intensive mentorship to an MWH governance committee comprised of community-selected members. We then examined how different stakeholders perceive community ownership of the MWH.

**Methods:**

We conducted 42 focus group discussions with community stakeholders (pregnant women, fathers, elders, and community health volunteers) and 161 in-depth interviews with MWH stakeholders (health facility staff, district health officials, and MWH governance committee and management unit members) at multiple time-points over 24 months. We conducted a content analysis and triangulated findings to understand community ownership of the MWH and observe changes in perceptions of ownership over time.

**Results:**

Community members’ perceptions of ownership were related to their ability to use the MWH and a responsibility toward its success. Community and MWH stakeholders described increasingly more specific responsibilities over time. Governance committee and management unit members perceived their ability to represent the community as a crucial component of their role. Multiple respondent types saw collaboration between the governance committee and the health facility staff as key to allowing the MWH to meet its goal of serving the community.

**Conclusion:**

The perceptions of community ownership evolved as the intervention became more established. Use of the MWH, and clear understanding of roles and responsibilities in management of the MWH, seemed to foster feelings of community ownership. To improve the sustainability of community-based maternal and child health programs, interventions should be accessible to target communities and clear roles should be established among stakeholders.

## Abstract (precis)

Community-based maternal child health programs should foster a sense of community ownership to promote sustainability. In rural Zambia, health interventions should be accessible to target communities and clear roles should be established among stakeholders for effective governance.

## Teaser

**Key Findings:**

- Community members’ perceptions of ownership were related to their ability to use the maternity waiting home (MWH) and feeling a sense of responsibility toward its success.
- Representing their community’s interests was a crucial component of the role of the governance committee and management unit.
- Collaboration between the governance committee and the health facility staff was key to allowing the MWH to meet its goal of serving the community.

**Key Implication:**

- To improve the sustainability of community-based maternal and child health programs, program managers should ensure that interventions are accessible to target communities and clear roles are established among stakeholders.

## INTRODUCTION

To address the underlying causes of maternal mortality and morbidity, governments throughout sub-Saharan Africa (SSA) have implemented both supply-side and demand-side interventions.^1^^,^^2^ Many interventions build capacity of community members through community health worker training^1^^,^^2^ or use community volunteers to conduct health education and promotion activities.^3^^,^^4^ In Zambia, community members have been engaged in the implementation of maternity waiting homes (MWHs),^4^^–^^6^ which are residential dwellings located near heath facilities where women can stay to await childbirth by a skilled birth attendant and receive postnatal care services. Women who can access a health facility with a high-quality MWH are more likely to deliver at a facility where a skilled birth attendant is present.^6^ Additionally, this type of intervention has shown promising results on reducing mortality among pregnant mothers in Africa.^7^ However, in several studies of MWHs, women and community members were concerned about the sustainability of the intervention, regardless of its perceived success.^4^^,^^8^^,^^9^

Evaluating the sustainability of externally funded interventions to address maternal mortality is increasingly important in global health.^10^^,^^11^ A recent review found that very few health interventions in SSA examined sustainability outcomes.^12^ Of those that did, the majority identified community ownership and mobilization as crucial facilitators of intervention sustainability.^12^ For example, an evaluation of the large-scale, comprehensive Saving Mothers Giving Life initiative, which aimed to rapidly reduce maternal mortality in Zambia and Uganda from 2012 to 2016 through community health worker mobilization, doctor and nurse training, and facility upgrades,^13^ found that the intervention’s ability to foster a sense of local ownership around the intervention was an essential factor in the early maintenance of its gains in maternal and child health outcomes.^14^

The 2005 Paris Declaration on Aid Effectiveness and the 2008 Accra Agenda for Action both argue in favor of a country defining its own development priorities and designing and leading programs promoting these priorities.^15^ The need for country or community ownership of health interventions, for example through sustained government or ministry of health funding or community contributions, is rooted in ideological values of self-determination and has been posited to be an effective approach to sustainability.^16^^–^^18^ The concept of community ownership emerged in the literature several decades ago from similar ideological origins.^19^ In the health context, community ownership has been defined as “community leaders’ levels of perceived control over key functions of a [health program] at the time of measurement.”^20^^,^^21^ Ultimately, empowerment is the desired outcome, where communities have control and decision-making ability over these health interventions and their future, whether at the local (community ownership) or national level (country ownership).^22^

When conducting formative research to design a community-driven MWH model in rural Zambia, we found that community members considered community ownership essential to the success of a potential MWH intervention.^8^ More specifically, community members linked the concept of sustainability of the MWH intervention to local or community ownership but did not offer clear definitions or examples of what ownership meant to them.^8^^,^^23^ In the evaluation of the sustainability of our MWH model in rural Zambia,^8^^,^^9^^,^^23^ we used qualitative methods to explore local perceptions of community ownership over the course of the MWH intervention. This article qualitatively explores how different stakeholders perceived community ownership of the MWH and how this changed over the first 24 months of MWH operations.

We used qualitative methods to explore local perceptions of community ownership of MWHs and how they changed over time.

## METHODS

### Study Setting/Intervention Design

The Maternity Homes Access in Zambia project constructed 10 MWHs adjacent to rural health centers able to provide obstetric care for uncomplicated deliveries and within 2 hours of time to a referral hospital equipped to care for women experiencing obstetric complications. The intervention was implemented in 4 districts of rural Zambia: Choma, Pemba, and Kalomo (in Southern Province) and Nyimba (in Eastern Province). All study districts are primarily rural with some peri-urban pockets. Choma has 247,860 people, 76% of whom live in rural areas. At the time of the 2010 census, Pemba was part of Choma. Kalomo has 258,570 people, most of whom live in a rural area (93%).^24^ Nyimba has 77,359 people, 91% of whom live in rural areas.^24^

We gathered community input from community members and relevant stakeholders in the health system and traditional leadership structures to design an intervention that would meet community standards of acceptability.^4^^,^^8^^,^^9^ The resulting 3-pillar conceptual model (core MWH model) focused on: (1) the establishment of quality MWH structures with functional infrastructure and amenities; (2) the need for a community-based system to oversee the daily management, finances, and future maintenance requirements of the MWHs without overburdening the existing health system; and (3) the need to be linked with the health system for clinical care of waiting women and education. The core MWH model met cultural-appropriateness and was aligned with Ministry of Health policy.^8^^,^^9^^,^^23^

In accordance with the management pillar, we engaged stakeholders at all levels of the MWH ecosystem before and during the intervention implementation (Figure). We engaged community members, including traditional leadership (i.e., chiefs and the village headmen who represent the chiefs), to sensitize them on the benefits of an MWH, actively participate in the governance and management of the MWH through selected community members, and contribute to the financial and operational sustainability of the MWH. We also engaged health system staff, which included staff at the health facility and district health office levels, to ensure our goals were aligned. For example, we engaged district health staff to participate in steering committees to advise the creation of the MWH governance committees and MWH management units. We engaged health facility staff and community health outreach workers to actively participate in the governance and management of the MWHs and to ensure linkage of the MWH to the facility.

**FIGURE.**
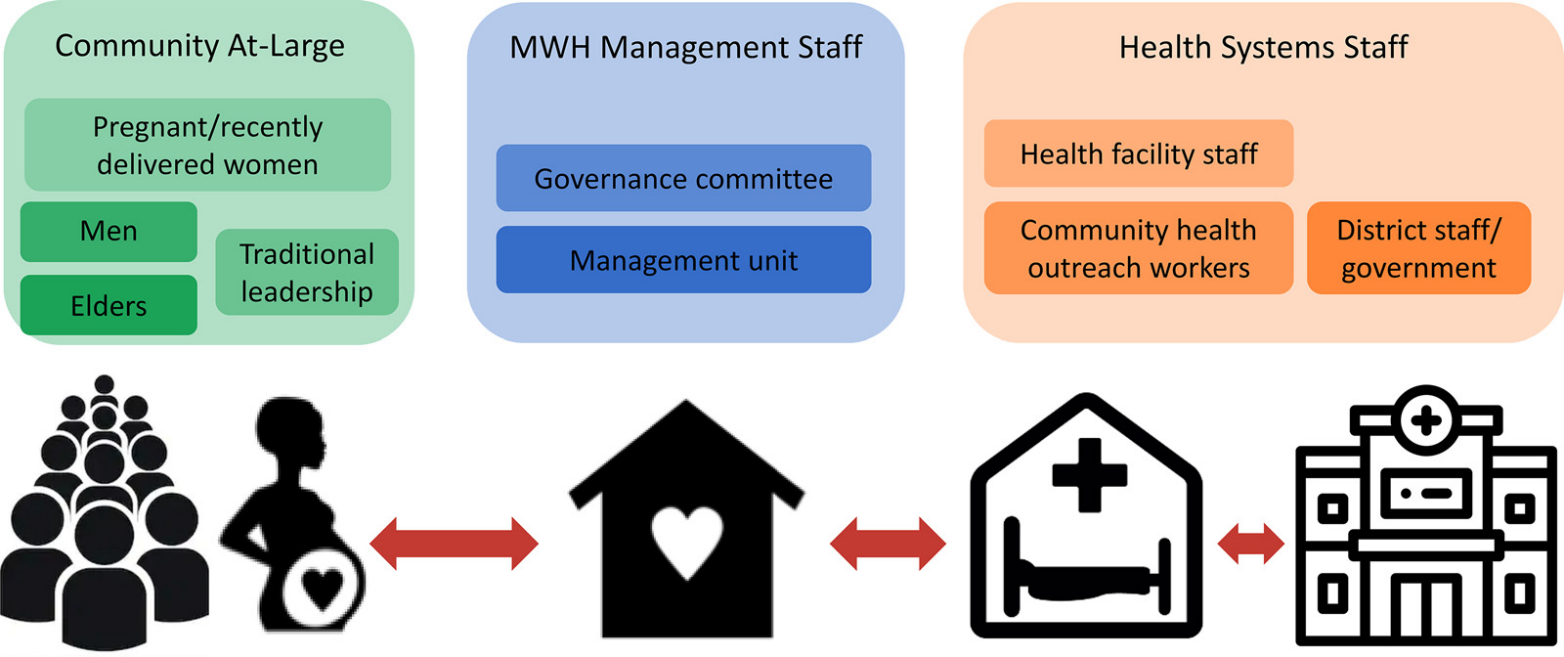
The Maternity Waiting Home Ecosystem in 4 Districts in Zambia

We provided training and ongoing mentorship to community-elected MWH governance committees and management units (GCMU). The governance committees are comprised of community members and health facility staff. The management units are comprised of community members or health facility staff selected by the governance committees. The governance committee is responsible for managing the MWH, mobilizing resources, and overseeing the management unit to ensure sustainability of the MWH. The management unit is responsible for the daily operations of the MWH and management of MWH assets. Additionally, we covered the start-up costs for community-led income-generating activities that could help support costs associated with the MWH and contribute to its financial sustainability. The project phased out supporting implementation in April 2018 but continued to monitor intervention activities through October 2018. The evaluation of the implementation of the intervention has been described elsewhere.^25^

### Thematic Framework

To evaluate the sustainability of our MWH intervention, we relied on findings from the formative evaluation and Scheirer and Dearing’s framework for the sustainability of public health programs.^26^ The framework determines sustainability by asking: (1) whether program activities were continued after external support ends, (2) whether community-level partnerships or coalitions developed during the funded program were maintained, and (3) whether new organizational practices, procedures, and policies that were started during program implementation were maintained. We hypothesized that community ownership may be an important mediator of these constructs and therefore an essential component of sustainability. This hypothesis was in line with findings from our formative work, which qualitatively underscored the importance of ownership of the MWH intervention by the community.^4^^,^^8^^,^^9^ We deliberately did not define community ownership, but rather allowed our stakeholders to explain ownership in their own words.

### Data Collection and Management

We conducted 42 focus group discussions (FGDs) and 161 in-depth interviews (IDIs). The FGDs were conducted with 412 community members (14 groups of pregnant or recently delivered women, 10 groups of men with a child under age 1, 9 groups of community elders, and 9 groups of community health volunteers). Safe Motherhood Action Groups made up the majority of community health volunteers, but traditional birth attendants were also part of the FGDs. The IDIs were conducted with MWH governance committee and management unit members, and health systems staff (health facility staff, district health officials). FGDs were conducted at 3 timepoints: immediately following intervention launch (Octo-ber 2016 to January 2017); during the intervention (August 2017 to September 2017); and after implementation phaseout (April 2018 to May 2018). IDIs were conducted at 4 timepoints: immediately following intervention launch (October 2016 to January 2017); during the intervention (April 2017 to June 2017 and November 2017 to January 2018); and after implementation phaseout (July 2018 to October 2018). We used convenience sampling to select the most senior person available on the day of visit for the district staff, health facility staff, governance committee, and management unit IDIs. Community health volunteers recruited FGDs participants from varying distances from the health facility. Both qualitative instruments captured basic demographics and had questions that elicited perceptions of the MWH operations and stakeholder roles as well as perspectives on health facility engagement, community ownership, and long-term sustainability.

Local data collectors fluent in English and the local languages, who were trained in qualitative interviewing techniques, the interview guides, and research ethics, administered the IDIs and FGDs. Data collectors were not members of the intervention implementation team, which provided direct mentorship and support to the GCMU, as described above. Data collectors participated in a refresher training before each round of qualitative interviews. Predefined probes were adapted and refined based on results from each previous round. IDIs and FGDs were audio recorded, translated into English, and transcribed verbatim into Microsoft Word.

### Analysis

Transcripts were systematically coded in NVivo version 11 (QSR International). The main coding nodes were identified *a priori* based on the questions and probes in interview guides. Transcripts were double coded against the theoretical framework and to a topic or theme. Additional nodes were added as themes emerged during coding. We conducted a content analysis to assess respondent definitions of community ownership and applicability to the MWH intervention among respondent types and over time.^27^

Demographic data were captured in Survey-CTO Collect version 2.212 (Dobility, Inc.) and analyzed in SAS version 9.4 (SAS Institute Inc.). Proportions were calculated for respondent sex, occupation, and school attendance. Means and standard deviations (SD) were calculated for respondent age and highest grade completed. We had missing data (n=24) for years of education for elders and community health volunteers at project phaseout.

### Ethics

We obtained ethical approval through the Boston University Medical Campus Institutional Review Board and the ERES Converge Institutional Re-view Board in Lusaka, Zambia, and approval by the Zambian National Health Research Authority. Written informed consent was obtained from respondents in the language they were most comfortable using: English, Chinyanja, or Chitonga.

## RESULTS

We have provided a description of IDI respondents (Table 1) and FGD respondents (Table 2). Results are presented by the 2 main themes that respondents discussed: (1) general perceptions of ownership of the MWH, and (2) roles and responsibilities for each stakeholder toward the MWH.

**TABLE 1.** Demographic Characteristics of Focus Group Discussion Respondents (N = 412) on Ownership of Maternity Waiting Homes in 4 Districts in Zambia

	ImmediatelyPost-launch	**ImplementationPeriod**	**ProjectPhaseout**	**Total**
	October 2016 toJanuary 2017	August 2017 toSeptember 2017	April 2018 toMay 2018	
**Pregnant/recently delivered women**	n=46	n=34	n=40	n=120
Age, y, mean (SD)	26 (7)	25 (6)	25 (6)	25 (6)
Pregnant, No. (%)	20 (43.5)	19 (55.9)	22 (55.0)	61 (50.8)
Education, y, mean (SD)	7 (3)	7 (3)	–	7 (3)
Parity, mean (SD)	3 (2)	3 (2)	3 (2)	3 (2)
Gravida, mean (SD)	3 (2)	3 (2)	4 (2)	3 (2)
Married/cohabitating, No. (%)	37 (80.4)	28 (82.4)	32 (80.0)	97 (80.8)
**Men with child under 1 year old**	n=46	n=36	n=16	n=98
Age, y, mean (SD)	33 (12)	34 (9)	30 (9)	33 (11)
Education, y, mean (SD)	8 (3)	9 (3)	5 (4)	8 (3)
Number of biological children, mean (SD)	5 (3)	5 (3)	4 (2)	4 (3)
Married/cohabitating, No. (%)	46 (100.0)	35 (97.2)	14 (87.5)	95 (96.9)
**Elders**	n=46	n=38	n=16	n=100
Female, No. (%)	29 (63.0)	17 (44.7)	9 (56.3)	55 (55.0)
Age, y, mean (SD)	79 (20)	63 (9)	64 (9)	70 (17)
Years of education, y, mean (SD)	5 (4)	6 (4)	–	5 (4)
Number of biological children, mean (SD)	7 (3)	8 (4)	7 (3)	7 (3)
Married/cohabitating, No. (%)	34 (73.9)	27 (71.1)	10 (62.5)	71 (71.0)
**Community health volunteers^a^**	n=46	n=40	n=8	n=94
Female, No. (%)	23 (50.0)	29 (72.5)	5 (62.5)	57 (60.6)
Age, y, mean (SD)	53 (19)	44 (10)	46 (11)	49 (16)
Education, y, mean (SD)	9 (2)	9 (2)	–	9 (2)
Number of biological children, mean (SD)	7 (2)	5 (3)	4 (3)	6 (3)
Married/cohabitating, No. (%)	35 (76.1)	25 (62.5)	6 (75.0)	66 (70.2)

Abbreviation: SD, standard deviation.

a Safe Motherhood Action Group.

**TABLE 2.** Demographic Characteristics of In-depth Interview Respondents (N=161) on Ownership of Maternity Waiting Homes in 4 Districts in Zambia

	**Immediately Post-launch**	**Implementation Period**		**Project Phaseout**	**Total**
	October 2016 to January 2017	April 2017 to June 2017	November 2017 to January 2018	July 2018 to October 2018	
**Management unit (MWH staff)**	n=8	n=10	n=10	n=9	n=37
Female, No. (%)	8 (100.0)	8 (80.0)	6 (60.0)	9 (100.0)	31 (83.8)
Age, y, mean (SD)	41 (15)	39 (14)	39 (12)	37 (10)	39 (13)
Education, y, mean (SD)	10 (2)	10 (2)	10 (2)	10 (2)	10 (2)
Farmers, No. (%)	4 (50.0)	9 (90.0)	4 (40.0)	8 (88.9)	25 (67.6)
**Governance committee (MWH staff)**	n=17	n=18	n=16	n=10	n=61
Female, No. (%)	11 (64.7)	9 (50.0)	10 (62.5)	5 (50.0)	35 (57.4)
Age, y, mean (SD)	50 (5)	47 (8)	49 (7)	49 (13)	49 (8)
Education, y, mean (SD)	9 (2)	10 (2)	9 (2)	10 (2)	9 (2)
Leadership position in governing committee, No. (%)	5 (29.4)	10 (55.6)	10 (62.5)	9 (90.0)	34 (55.7)
Farmers, No. (%)	16 (94.1)	17 (94.4)	13 (81.3)	8 (80.0)	54 (88.5)
**Health facility staff**	n=11	n=10	n=10	n=10	n=41
Female, No. (%)	5 (45.5)	6 (60.0)	3 (30.0)	2 (20.0)	16 (39.0)
Facility in-charge, No. (%)	7 (63.6)	4 (40.0)	2 (20.0)	6 (60.0)	19 (46.3)
Clinical position, No. (%)					
Clinical officer	1 (9.1)	1 (10.0)	2 (20.0)	1 (10.0)	5 (12.2)
Nurse/midwife	6 (54.6)	7 (70.0)	2 (20.0)	2 (20.0)	17 (41.5)
Non-skilled birth attendant staff	1 (9.1)	1 (10.0)	3 (30)	2 (20.0)	7 (17.1)
Years working in the health system, mean (SD)	14 (10)	10 (7)	6 (8)	10 (8)	10 (8)
**District health officers**	n=6	n=9	n=3	n=4	n=22
Female, No. (%)	2 (33.3)	3 (33.3)	1 (33.3)	1 (25.0)	7 (31.8)
District Health Officer, No. (%)	3 (50.0)	0 (0.0)	0 (0.0)	0 (0.0)	3 (13.6)
Years working in the health system, mean (SD)	11 (7)	11 (5)	9 (6)	16 (4)	12 (6)

Abbreviations: MWH, maternity waiting home; SD, standard deviation.

We conducted 42 FGDs with 412 individuals over 24 months (Table 1). FGD respondents were fairly similar across time points. Most had attend-ed at least some schooling although community elders had less than other respondents. Comm-unity health outreach workers and community elders were more likely to be male. Pregnant and recently delivered women were slightly younger and had experienced 3–4 live births, and men had 4–5 children.

Within the health system and MWHs, 161 IDIs were conducted (Table 2). The majority of MWH staff (management unit and governance committee) interviewed were female and the majority of health system staff respondents were male. The health system staff had been in their current positions a few years, working in the broader health system for much longer.

### Theme 1: General Perceptions of Ownership

Generally, respondents agreed at all time points that the community had an important ownership role in the MWH. However, community members (FGD respondents) described ownership from the point of view of potential or real users and MWH stakeholders (IDI respondents), who are part of the health system, described ownership in terms of roles and management.

FGD respondents perceived that they—the community—owned the MWH and described 2 different elements of ownership: (1) the ability for any member of the community to use the MWH, and (2) a sense of responsibility for the future success of the MWH. A sample of illustrative quotes are included in Supplement 1. Across all time points, respondents justified that the MWH belonged to the community because any member could use it during their pregnancy. Specifically, pregnant women talked about being able to stay in the MWH and use its amenities, therefore being the owners of the MWH:

*The MWH is for every person, but to be specific, the owners are the pregnant women because they are the ones that use it.* —Pregnant woman, Project phaseout

Many stated that not having cost associated with usage made them feel like they owned it. Others reported that not experiencing discrimination fostered a sense of ownership.

Although all community members emphasized the importance of being able to use the MWH, in particular, men, community health volunteers, and elders mentioned a dimension of responsibility for the MWH as they described ownership. Specifically, men and community health volunteers described needing to look after or take care of the MWH:

*It is ours in the sense that the users are the community, so it’s the community’s responsibility to take care of it. If anything gets damaged it’s the community to take care of it.* —Man, Implementation period

Elders described that having made a financial contribution as a community to the initial construction of the MWH bolstered their sense of ownership because money came from the community to maintain the MWH.

*Because we suffered to build it, we are supposed to take care of it. Because everyone took part in the building of the MWH, everyone feels it belongs to them. So taking care of it, everyone is ready to do that.* —Elder, Immediately post-launch

In the IDIs, GCMU respondents consistently described the MWH as belonging to the community and/or pregnant women. Over time, GCMU respondents also better articulated what specifically was being “owned” and better described their own roles and responsibilities in relation to the MWH. Respondents increasingly described material assets and IGA revenue as belonging to the community, but explained that it was earmarked for the care of the MWH or pregnant women and managed by the governance committee.

District staff discussed the MWH as belonging to both the community and the health facility from the outset:

*[The MWH] is for the whole community in conjunction with the [health] facility. The greater part of the ownership is [shared] by the community because they’ve been involved in construction, even bringing materials, and when they were being launched. The community was involved, so they know that this is our structure, because it’s built for us.* —District health officer, Immediately post-launch

Health facility staff’s perceptions evolved over time. Although health facility staff discussed shared ownership of the MWH with the community, during later rounds of interviews, they more clearly articulated the role of the health facility, culminating with the MWH being described as an extension of the health facility:

*The MWH is part of the clinic. When it came to electrification, we were using the same meter. We are even using the same water.* —Health facility staff member, Project phaseout

### Theme 2: Stakeholder Roles and Responsibilities

FGD and IDI respondents described the specific MWH-related roles for each stakeholder with increasing specificity over time (Table 3). Respond-ents generally agreed on the role of each stakeholder, but some nuances emerged for the roles of the community at large and the district health staff. Illustrative quotes are summarized in Supplement 2.

**TABLE 3.** Focus Group Discussion Respondents’ Perspectives on Stakeholders’ Maternity Waiting Home Roles and Responsibilities Over Time in 4 Districts in Zambia, October 2016 to October 2018

		**Immediately Post-launch**	**Implementation Period**	**Project Phaseout**
Community at-large	Community members	Need to contribute money	Pregnant women help with cleaning of MWH Community members at-large are responsible for maintenance and safety of MWH as well as contributing money, food, and building materials Traditional leadership is responsible for mobilizing contributions	Pregnant women help with cleaning of MWH and contribute to IGAs Community members at-large are responsible for maintenance and safety of MWH as well as contributing money, food, and building materials. Traditional leadership is responsible for mobilizing contributions
MWH Management Staff	Governance committee	Representative of communities Partner with health facility	Representative of communities Responsible for MWH management	Representative of communities Responsible for MWH management Communicate with traditional leadership Partner with health facility
	Management unit	Representative of communities	Representative of communities Help maintain the cleanliness and comfort of the MWH	Representative of communities Take care of day-to-day MWH needs
Health Systems Staff	District staff	Respond to health facility needs, but not MWH needs	Respond to health facility needs, but not MWH needs	Respond to health facility needs, which sometimes include MWH needs
	Health facility staff	Provide cleaning supplies Work with GC Provide clinical care Check in on mothers at the MWH	Partner with the GCMU for MWH management Provide clinical care Check in on mothers at the MWH Communicate with the district	Partner with the GCMU for MWH management Some participate in GC Provide clinical care Check in on mothers at the MWH

Abbreviations: GC, governing committee; GCMU, governing committee/management unit; MWH, maternity waiting home.

#### Role of the Community

Traditional leadership played an increasingly important role in the functioning of the MWH within the community over time. At the launch of the program, community health volunteers mentioned that the role of the village headmen was to regularly check on the MWH on behalf of the chiefs. Other respondent types such as the governance committee, health facility staff, and community members described traditional leadership as responsible for mobilizing community contributions for the MWH (described below) based on requests from the governance committee (Table 3).

The GCMU members highlighted that the community was the primary owner of the MWH and described that the community at large was responsible for making cash and/or in-kind contributions to the MWH to support maintenance. Community members felt they had a responsibility to make cash and/or in-kind contributions to the MWH:

*[Those of us] who come here, we do the cleaning on our own … just the way we do it back at our home.* —Pregnant woman, Immediately post-launch

Health facility staff and the GCMU felt the primary role for pregnant women was to use the MWH and sometimes assist with cleaning tasks. They also felt the community at-large should participate in the structural maintenance of the MWH.

Community members only vaguely described community contributions at the launch of the intervention but became increasingly specific over time. Pregnant women described having some responsibility toward helping keep the MWH clean. Respondents from the community described how community contributions of cash, food, and building materials ensured sustainability. However, many pregnant women said that community contributions were not always happening as planned, whether monetary or in kind, and expressed concern over the future of the MWH.

Finally, the GCMU and health facility staff described that the community had a mandate to ensure the future of the MWH. For example, health facility staff described that the community had control over the selection of governance committee members, which were in turn responsible for the success of the MWH. Therefore, the community owns the MWH but delegates its role to the governance committee:

*The community is there to ensure that their structure is community-driven. They should ensure, because the governance committee is chosen by the community, so the community will oversee whether those people are doing what is expected. They are monitors to ensure that the MWH is there. If a member of the governance committee is not working well, we’ll call the community and plan on how they can help the member or the committee or they can change the committee. The community has the mandate to change the committee because this is a structure that is going to benefit the community. These mothers that are coming here are coming from the villages and the community. The community is going to enjoy and should not shun coming to deliver from the clinic because of the conditions that are not good here.* —Health facility staff member, Project phaseout

#### Role of the Governance Committee and Management Unit

Over time, all respondent types described managerial and custodial roles for both the governance committee and management unit with increasing specificity (Table 3). At the launch of the intervention, most respondent types failed to describe clear structures for the committees. Later, governance committee members, health facility staff, and community members described the governance committee as responsible for managing the income of the MWH and for communicating with the village headmen about the MWH’s needs and possible community contributions. The management unit members described themselves as responsible for the day-to-day operations of the MWH:

*It is on me as the management unit because I am always here at the MWH and take care of this property on a daily basis, give reports on what is damaged and anything that needs improvement. If I don’t do so, then the MWH will be vandalized, and it will not last long.* —Management unit staff member, Project phaseout

Community members highlighted the role of the management unit as responsible for explaining the rules of the MWH to expecting mothers and ensuring they felt welcome upon arrival.

Governance committee members described a hierarchical relationship between themselves and the management unit. The management unit is responsible for escalating MWH issues to the governance committee when needed; however, no exact mechanism was described by GCMU members. Overall, all respondent types described the GCMU members as representatives of the community who work as partners to manage the MWH. Additionally, GCMU respondents perceived themselves and the committee structures as important representatives of the community.

*We are the ones who are supposed to see to it that all is working accordingly because this MWH belongs to us. If there is anything happening, we communicate with the rest of the community to inform them.* —Governance committee member, Implementation Period

Health facility staff referred to governance committee members as custodians of the MWH, including its assets and IGA-generated income, on behalf of the community during later rounds of interviews. In essence, the community “owns” the MWH and the governance committee, which is made up of community-selected members to represent the interests of the community with respect to the MWH operations. Ultimately, governance committee members considered themselves responsible to the community for achieving the mission and goals of the MWH by providing pregnant women with a high-quality MWH, but indicated a reliance on the management unit and the community at-large.

#### Role of the Health Facility Staff

During the implementation of the project, all respondent types described health facility staff as having a clinical role in caring for the pregnant women utilizing the MWH. They regularly visit the pregnant women to check on their health and monitor potential pregnancy-related complications.

*The responsibilities we have mainly concern the mothers. There are some who have overstayed, so we go through the antenatal bookings they have attended [and if we find they were] not fully examined, we will go through that to see if our findings are okay. We refer them together with the management to a higher-level hospital. Apart from that, we still encourage them if there are any questions or problems. We ask them to still come because it is, we are still one facility.* —Health facility staff member, Implementation period

Many health facility staff echoed this description and one gave the example of having midwives checking on expecting women at the MWH while the GCMU ensured the MWH remained clean.

Over time, the role of the health facility staff evolved into a supporting role for the GCMU (Table 3). At the launch of the intervention, the health facility staff expressed that they felt responsible for providing the MWH with cleaning supplies because the MWH was not yet generating income. Gradually, and as the income-generating activities were implemented, the management and operations of the MWH were assumed by the GCMU, in collaboration with the health facility. Community members, including recently delivered women, provided a similar description of the evolution of the health facility staff’s role over time. At the launch of the intervention, community members described health facility staff such as nurses, as responsible for the management of the MWH. Over time, respondents began to describe the GCMU and the health facility staff as partners in the operations of the MWH meant for the community. The district staff described that the health facility staff were also responsible for communicating with the district about the MWH’s needs.

#### Role of the District Staff

Over time, the district staff consistently described themselves as playing a supervisory role based on health facility requests (Table 3). District staff highlighted their need to be responsive to financial and operational issues of the health facility and the MWH but offered no specifics. For example, a district staff member explained that the district staff could assist with providing resources to help the MWH operations continue, but did not specify what might warrant their assistance.

*I think one of our major roles here at the district is to help the facility and the community to handle some of the major problems that they face that they are not able to handle at their level and maybe to source support from others or maybe provide resources which can help them run. There are issues which they can handle on their own but there are issues which may need external support, which I think the district should be key in coordinating that area.* —District staff member, Implementation period

No other respondent type, including the health facility staff and governance committee, described a role for the district staff in the operations of the MWH.

## DISCUSSION

This study qualitatively explored community ownership of MWHs from the perspective of multiple stakeholders over 24 months, from launch of the intervention to after external support for the program had ended. A core MWH model was designed in consultation with local stakeholders, community leaders, and community members throughout Zambia.^4^^,^^8^^,^^9^^,^^23^^,^^25^ During these consultations, participants acknowledged the need for communities to contribute to the operations and maintenance of the MWH and stated that community involvement or “community ownership” was crucial to MWH sustainability, which has been corroborated by studies in other areas.^9^

Community ownership is a challenging concept to define because different stakeholders bring varying perspectives and the term is often conflated with other concepts related to community engagement or sustainability.^22^ When asked about who owned the MWH, respondents all agreed that the MWH belonged to the community, but differed in how and what they described as “ownership.” Although respondents in the community described ownership in terms of the MWH being available to them and used by community members, respondents within the health system linked ownership to responsibility, similarly to what has been reported in the literature by implementers of other community-driven interventions.^28^

Community members focused on describing how they felt the MWH belonged to them because the MWH was built for them. Specifically, community members emphasized the importance of everyone being able to use the MWH, especially because it was free of charge. This finding is interesting considering the country-wide decision to eliminate user fees for maternal health services in Zambia, even if existing evidence does not clearly link the absence of fees with increased utilization.^29^^–^^31^ Our results indicate that not having to pay a fee to use the MWH may have influenced community members’ decision to participate in the intervention and was an important determinant of whether community members felt a sense of ownership over the MWH.

Although the first finding gives insight into what may be a necessary component to foster a sense of community ownership, our second finding was that respondents connected the concepts of community ownership and sustainability with stakeholder roles and responsibilities. Rather than describing their perception of the MWH from the point of view of a user or potential user, respondents who were involved in the operations and management of the MWH focused on how the role of each stakeholder was essential to the overall functioning of the MWH and its future sustainability. Over time, respondents increasingly described more specific roles for other stakeholders.

Not having to pay to use the MWH may have influenced community members’ decision to participate in the intervention and determined whether community members felt ownership over the MWH.

The role of the community became more precise over time. At first, the community was expected to contribute, but the nature of the contributions and the mechanism through which contributions could be made remained vague. At later timepoints, respondents described that the community was responsible for making cash and/or in-kind contributions. These contributions were to be mobilized from community members by traditional leadership based on feedback from the governance committee.

The district staff were responsible for supervising the health facility staff, but the health facility staff, governance committee, and management unit were essential to the functioning of the MWH. Specifically, the health facility provided clinical care to pregnant women, supported the GCMU, and communicated with government-level actors such as the district staff. The GCMU had different roles in the operations of the MWH, with the governance committee playing a managerial role in the MWH and the management unit being responsible for the day-to-day activities of the MWH. Members of both groups were considered representatives of the community. As such, the concepts of community ownership and clear roles for the stakeholders involved in the management of the MWH were linked together by the respondents.

While respondents initially discussed ownership of the MWH as falling to either the community or the community and the health system, when probed further, specific roles and responsibilities for stakeholders at multiple levels were identified by all respondents as critical to the long-term success of the MWH. The description of these roles with increasing specificity over time is interesting, as respondents were able to articulate responsibilities and things that are needed for the MWH to function now and in the future as they gained more experience managing and using the MWH. This increasing specificity is to be expected as the MWH operated for nearly 2 years at the time of the last data collection point. All roles became clearer to the stakeholders involved, not only their own roles but also the roles of the other stakeholders. These specific roles and the emphasis on their importance persisted even when external financial and mentorship support from the project staff ended.

Other factors such as social context, financial support, and organizational partnerships affect the sustainability of the intervention, but the perspectives of our respondents are important because they illustrate how the concept of community ownership can be operationalized to serve the purpose of sustainability. In summary, users described ownership as an ability to stay at the MWH and benefit from it, whereas stakeholders involved in the operations of the MWH described ownership as a well-established set of mechanisms where each stakeholder had a specific role widely known to other stakeholders.

Respondents within the community and within the health system described that the MWH intervention created an ecosystem of shared responsibility around the current and future functioning of the MWH (Figure). The GCMU’s roles were to manage the operations of the MWH in collaboration with the health facility, and that they had a responsibility toward the community to ensure that the MWH was providing quality services and that its operation was sustainable. The collaboration between the GCMU and health facility staff represents a critical element of the intervention, the point at which the health facility staff accepted to assume a level of responsibility for the MWH and its functioning, including through communication with the district. This shared responsibility between the community-driven GCMU and the health facility staff reduces the managerial burden of the staff and allows for focus on their other duties. In previous findings, health system stakeholders had similarly described the importance of the GCMU because its presence allows health system staff to attend to their clinical duties.^32^ Although the community representatives on the GCMU are the instrumental link to the community, the health facility staff is the instrumental link to the district and government-level actors. This governing body where community representatives and health facility staff formally collaborate to ensure the smooth operations of the MWH serves as a key opportunity to connect the community with the formal health system.

Existing literature indicates that social accountability is an important factor of community participation, especially to ensure equity and a high level of quality in service provision.^33^ This type of accountability allows for the successful collaboration of stakeholders, which is the case here. One caveat of this concept is that if community participation does not yield positive results, community members may become less engaged.^33^ In our setting, respondents unfortunately did not describe clear mechanisms of accountability between the governance committee and the community, or the governance committee and the management unit, or the GCMU and the health facility. While respondents gave some examples of how these stakeholders communicate, they did not give many examples of successful conflict resolution. In that regard, the lack of described mechanisms for feedback is worrisome. Surpris-ingly, respondents did not see the annual meetings held by the GCMU with community members as a mechanism for raising and resolving issues.^34^ However, this may be the result of not probing for examples of conflicts during the IDIs and FGDs or because interviews and FGDs took place too soon after external support had ended for serious concerns to have emerged. It is also possible that the respondents interviewed in our sample were not among the annual meeting participants. Consensus around roles and responsibilities of members is essential for governance models like the GCMU or health facility committees.^35^ Our results add to the evidence showing a link between specified roles and effectiveness of a health intervention.^35^ However, our data do not allow us to fully understand how these roles will continue to be maintained, especially when conflicts inevitably arise. This represents an area of both improvement and exploration, as other studies have elicited the difficulties of understanding what works and does not work after external support is removed in interventions that involve communities and community representation at the facility level.^11^^,^^35^^,^^36^

During the design of this intervention, community respondents had stated that community ownership was essential to the sustainability of the intervention, but they did not provide clear parameters for ownership. What emerges from our findings is that community representation through a structure like the governance committee may be an adequate response to the community’s demand for community ownership. The governance committee is concerned with representing the interests of community members, who themselves identified ease of access to the MWH as an essential component of ownership. Through the core MWH model, the governance committee and the health facility staff became partners in the operations of the MWH, with each party able to facilitate communication with the community and the district about MWH needs. The health facility staff was a link to the formal health system, whereas the GCMU served as a link to the broader community surrounding the MWH.

Participation of the most marginalized members of the community in these representative processes is a concern. Many interventions have sought to improve the effectiveness and sustainability of health programs by engaging community members, especially those directly affected by the health programs.^37^ Our intervention was able to achieve broad engagement with the community at large, including our target population of pregnant/recently delivered women. Unfortunately, our findings do not provide information on whether the poorest, most vulnerable pregnant women within the MWH catchment areas felt the same amount of ownership as those who might be considered less vulnerable. Further studies should continue to explore which strategies work best to ensure equity within processes that aim to increase community participation^38^ and ownership perhaps by focusing on who is chosen to represent the voice of the community. Further work examining the composition of the GCMU has been published elsewhere.^34^

It is also worth noting that even though respondents were blind to the official outcomes, such as district-wide skilled birth attendance rates, many respondents perceived the intervention to have had positive outcomes on maternal mortality and have been beneficial to the community.^32^ We hypothesize that the sense of ownership may be stronger if the intervention is perceived to have a positive impact on maternal child health outcomes because community members would want to sustain these effects and the MWH model.

We had posited that the intervention would continue after the end of the external support period, in part due to the project’s goal of fostering community ownership. Respondent comments indicated that community ownership is connected to sustainability. Community members felt confident that the MWH was built for them and that they were able to use it. The community has a certain level of responsibility for the success of the MWH, but not necessarily sole responsibility. For the MWH to be sustainable, key roles need to be filled by different stakeholders. Specifically, the collaboration between the health facility and the GCMU is essential for the MWH to function well. They represent a linkage between those running the MWH and the community that must exist for a sense of community ownership to emerge and is viewed as an essential component of sustainability by respondents involved in the management of the MWH.

### Limitations

There are several limitations with this analysis. First, our purposive sampling method, while allowing for a wider variety of opinions to be collected, may have resulted in over-representing the views of some groups such as women and farmers. However, our analysis of these perceptions was conducted across multiple time points and several stakeholder types, which strengthens our findings. Second, we had limited ability to explore some of our findings in greater depth. For example, our data do not allow us to know reasons for the lack of detailed mechanisms for conflict resolution or problem-solving within the MWH. Thirdly, project staff could have influenced the roles and responsibilities of stakeholders over time, through contact for project implementation and interviews themselves. These processes could have influenced the final outcome of how community ownership was expressed by respondents. However, because we did not use a pre-established definition for community ownership, we believe that our findings should not be overly impacted by this effect. Finally, our focus on community ownership emerged from our formative work, during which community members underscored the importance of this concept to the sustainability of a MWH intervention. We acknowledge that other populations may consider government ownership or other strategies as important routes toward sustainability.

## CONCLUSION

Considering the need to ensure the sustainability of maternal health interventions, we found it essential to assess how stakeholders understood the concept of community ownership of an MWH model. Community ownership has long been an ill-defined concept with little evidence for how it is operationalized on the ground. We found variation in the definition of ownership by stakeholder type. While users described ownership as an ability to stay at the MWH and benefit from it, stakeholders involved in the operations of the MWH focused on the importance of the collaboration between the governance committee and the health facility staff, which respectively represent the community at-large and the larger health system. These definitions and perceptions are particularly important to consider when designing health interventions to ensure that their positive impact continues once external support is withdrawn.

## Supplementary Material

20-00136-Fontanet-Supplement_1-clean.docx

20-00136-Fontanet-Supplement_2-clean.docx

## Notes

**Peer Reviewed**

**First published online:** August 17, 2020
