# Supplementary material for: A Qualitative Exploration of Community Ownership of a Maternity Waiting Home Model in Rural Zambia
Source: Glob Health Sci Pract. 2020 Sep 30;8(3):344–57. doi: 10.9745/GHSP-D-20-00136 (PMC7541113; doi:10.9745/GHSP-D-20-00136)
Supplement: 20-00136-Fontanet-Supplement_1-clean.docx [file 20-00136-Fontanet-Supplement_1-clean.docx]

**Supplement 1. Illustrative Quotes on Elements of Community Ownership**

| **Theme** | **Respondent Type** | **Quote** |
| --- | --- | --- |
| Ease of MWH use (equitable access, no fees) | Pregnant / recently delivered women | "The owner of this MWH is us who live in this area. For instance if I am not pregnant, I won’t have any work at this MWH but maybe my sister, aunt, or niece may come to use this MWH. So this MWH is ours, us who [live in this community]." (Implementation Period) |
|  |  | "The MWH is owned by those who stay there so it is the pregnant women that own the house." (Implementation Period) |
|  |  | "The MWH is for every person but to be specific the owners are the pregnant women because they are the ones that use it." (Project phase-out) |
|  | Men with child | “I remember when they were officially opening the MWH. Some people thought that maybe it was going to be a fee-paying MWH. But when they were told that it was free for anyone to use, people were very happy. And they actually felt the sense of ownership right there and then." (Immediately post-launch) |
|  | Elders | “I think this MWH is ours because we are the ones using it." (Project phase-out) |
|  | Community health volunteers | "Just the nature of them [pregnant women] coming to use this MWH. It means they are very free to come and stay here at the MWH and use it. That means they know that this is their MWH. So even when they come for antenatal check-ups they usually chat within themselves to say, “We need to go there and use that MWH. It is ours." (Immediately post-launch) |
|  |  | " Community means everyone because I can say the MWH belongs to [name of community health volunteer], it belongs to her when she is pregnant; when I become pregnant it will be mine. That is why it is said to belong to the community" (Project phase-out) |
| Responsibility for success | Men | "It is ours in the sense that the users are the community, so it’s the community’s responsibility to take care of it. If anything gets damaged it’s the community to take care of it." (Implementation Period) |
|  | Community health volunteers | "It is ours as a community, because we are the ones that can look after it. So it becomes for the community because we are the ones that can maintain it or destroy it" (Implementation Period) |
|  | Elders | “Because we suffered to build it, we are supposed to take care of it. Because everyone took part in the building of the MWH, everyone feels it belongs to them. So taking care of it, everyone is ready to do that" (Immediately post-launch) |
|  |  | "That MWH is ours in the community […] because the money used to do some things comes from us” (Implementation Period) |
|  | Governance Committee | " Knowing that (the MWH’s) assets are owned by everyone, there is a need to care for them." (Implementation Period) |
|  | District Health Officers | “(The MWH) is for the whole community in conjunction with the (health) facility. The greater part of the ownership is (shared) by the community because they’ve been involved in construction, even bringing materials, and when they were being launched. The community was involved, so they know that this is our structure, because it’s built for us.” (Immediately post-launch) |
|  | Health facility staff | “The MWH is part of the clinic. When it came to electrification, we were using the same meter. We are even using the same water. We didn’t have any challenges because (the MWH) is part of the clinic. (Project phase-out) |
