# Supplementary material for: A Qualitative Exploration of Community Ownership of a Maternity Waiting Home Model in Rural Zambia
Source: Glob Health Sci Pract. 2020 Sep 30;8(3):344–57. doi: 10.9745/GHSP-D-20-00136 (PMC7541113; doi:10.9745/GHSP-D-20-00136)
Supplement: 20-00136-Fontanet-Supplement_2-clean.docx [file 20-00136-Fontanet-Supplement_2-clean.docx]

**Supplement 2. Illustrative Quotes on Responsibilities of Stakeholders by Respondent Type**

| **Theme** | **Respondent Type** | **Quote** |
| --- | --- | --- |
| **Responsibilities** | | |
| Role of community | Pregnant / recently delivered women | “(Those of us) who come here, we do the cleaning on our own… just the way we do it back at our home.” (Immediately post-launch) |
|  | Men with child | “If something (at the MWH) is damaged, one of our local people can then do one or two things to fix it.” (Immediately post-launch) |
|  | Elders | “Yes, the community feels they have a sense of ownership over the MWH. They make contributions because we don’t want a situation whereby a window is broken then we can’t replace it.” (Immediately post-launch) |
|  | Elders | “The villages can help by making contributions, maybe a gallon of maize per village or household so the burden of food (at the MWH) can be lessened.” (Implementation period) |
|  | Community health volunteers | “The MWH is ours as a community because we are the ones that can look after it. We are the ones that can maintain it or destroy it.” (Implementation Period) |
|  | Governance committee | “ The mothers staying at the MWH come from the community and the people in the community have the mandate of guiding the house. The MWH is not for the governance committee or the health facility or the management unit, it is for everyone who is in the community.” (Implementation Period) |
|  | Pregnant / recently delivered women | “The community contributes towards the MWH. For instance, the church comes to clean the surroundings”. (Project phaseout)  “Yes the community helps with maintenance. The people from my community do come to slash, sweep and weed around the MWH.” (Project phaseout)  “(The other community members) are not together do not help (with weeding and maintenance).” (Project phaseout)  “They do not help though they are supposed to help.” (Project phaseout) |
|  | Men with child | “I think whenever there is need for any maintenance work at the MWH, the management unit must hold a meeting and summon the village headmen so that they can be told how much each village must contribute. This money can be used to repair beds, buy new mattresses. Once the village headmen have been informed, they can go back and inform their subjects to make contributions and use the money for maintenance of the MWH.” (Project phaseout) |
|  | Management unit | “It is us and the community who are responsible for the success of the MWH. An example of this would be building a needed kitchen. The community would have to organize building materials such as building sand, ferrying of river sand to the site, molding of bricks, and labor.” (Project phaseout) |
| Role of governance committee | Management unit | “It is in the hands of the health facility working hand-in-hand with the governance and the IGA, so that money can be used wisely and the MWH can be developed.” (Project phaseout) |
|  | Governance committee | “We are the ones who are supposed to see to it that all is working accordingly, because this MWH belongs to us. If there is anything happening, we communicate with the rest of the community to inform them.” (Implementation Period) |
|  |  | “Since there is the governance committee in place which stands in on behalf of the community, this governance committee is answerable to the community or to what is going on at the MWH, like the IGA. The community has that responsibility to know what is going on, whether the IGAs are making profit and how the MWH is benefiting or any other things that we are required to as expected.” (Implementation Period) |
|  | Health facility staff | “The community is there to ensure that their structure is community-driven. They should ensure, because the governance committee is chosen by the community, so the community will oversee whether those people are doing what is expected. They are monitors to ensure that the MWH is there. If a member of the governance committee is not working well, we’ll call the community and plan on how they can help the member or the committee or they can change the committee. The community has the mandate to change the committee because this is a structure that is going to benefit the community. These mothers that are coming here are coming from the villages and the community. The community is going to enjoy and should not shun coming to deliver from the clinic because of the conditions that are not good here.” (Project phaseout) |
|  | Pregnant women | “The nurses and the chairman [of the governance committee] that manages the MWH work together when a person who wants to deliver come they receive well and give her everything to use.” (Implementation Period) |
| Role of management unit | Management unit | “It is on me as the management unit, because I am always here at the MWH and take care of this property on a daily basis, give reports on what is damaged and anything that needs improvement. If I don’t do so, then the MWH will be vandalized and it will not last long.” (Project phaseout) |
|  | Pregnant women | “When you arrive here the MWH, the chair lady welcomes you and encourages you every morning to clean the surroundings happily.” (Project phaseout) |
| Role of health facility staff | Health facility staff | “The responsibilities we have mainly concern the mothers. We mainly go there to check on the mothers. There are some who have overstayed, so we go through the antenatal bookings they have attended [and if we find they were] not fully examined, we will go through that to see if our findings are okay. We refer them together with the management to a higher-level hospital. Apart from that, we still encourage them if there are any questions or problems. We ask them to still come because it is, we are still one facility.” (Implementation Period) |
|  |  | “The material assets of the MWH were donated, but the assets are owned by the community. We are the custodians, but the whole property is actually owned by the community. The clinic is part of that community.” (Implementation Period) |
|  | Governance committee | “This MWH is something that has really helped us a lot. Now, we have to come up with ideas on how to maintain this MWH by working together with the headmen, the community, the management unit, and the health center staff because this thing was not built for us but for the generations to come. (Implementation Period) |
| Role of district staff | District staff | “I think one of our major roles here at the district is to help the facility and the community to handle some of the major problems that they face that they are not able to handle at their level and maybe to source support from others or maybe provide resources which can help them run. There are issues which they can handle on their own but there are issues which may need external support, which I think the district should be key in coordinating that area.” (Implementation Period) |
